# Supplementary material for: Rapid glycemic regulation in poorly controlled patients living with diabetes, a new associated factor in the pathophysiology of Charcot’s acute neuroarthropathy
Source: PLoS One. 2020 May 21;15(5):e0233168. doi: 10.1371/journal.pone.0233168 (PMC7241699; doi:10.1371/journal.pone.0233168)
Supplement: S1 Fig — Values are given as median [interquartile ranges]. Statistical significance was tested with the non-parametric Friedman’s test. (DOCX) [file pone.0233168.s001.docx]

**Supplementary Figure 1.** Decrease in HbA1c levels before the onset of Charcot neuroarthropathy. Values are given as median [interquartile ranges]. Statistical significance was tested with the non-parametric Friedman’s test.
