# Supplementary figures and images for: Rapid glycemic regulation in poorly controlled patients living with diabetes, a new associated factor in the pathophysiology of Charcot’s acute neuroarthropathy
Source: PLoS One. 2020 May 21;15(5):e0233168. doi: 10.1371/journal.pone.0233168 (PMC7241699; doi:10.1371/journal.pone.0233168)

**Figure 2.** Reduction in HbA1c levels according to the type of diabetes


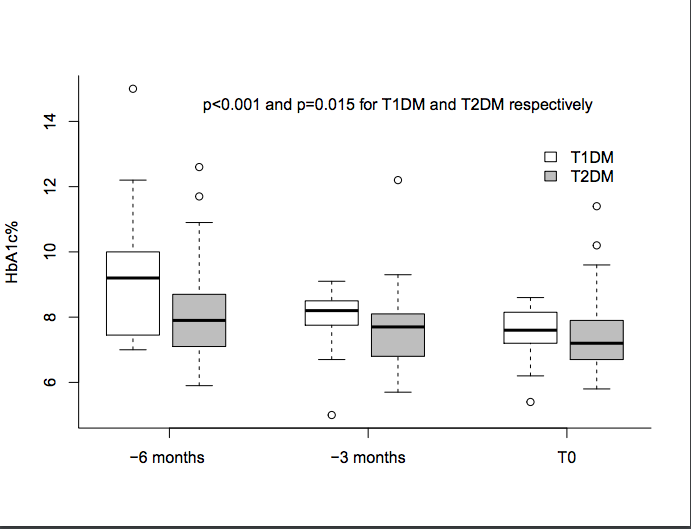

Supplement: S2 Fig — (DOCX) [file pone.0233168.s002.docx]
