# Supplementary material for: Rapid glycemic regulation in poorly controlled patients living with diabetes, a new associated factor in the pathophysiology of Charcot’s acute neuroarthropathy
Source: PLoS One. 2020 May 21;15(5):e0233168. doi: 10.1371/journal.pone.0233168 (PMC7241699; doi:10.1371/journal.pone.0233168)
Supplement: S3 Fig — (non parametric Friedman test). (DOCX) [file pone.0233168.s003.docx]

**Figure 3.** Heterogeneity of the magnitude of HbA1c reduction in the 6 months preceding the onset of Charcot neuroarthropathy. ( non parametric Friedman test)

**
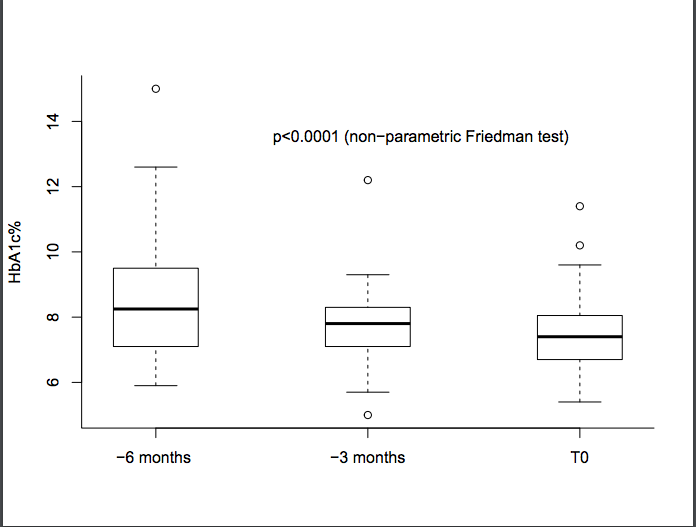
**
