# Supplementary material for: Rapid glycemic regulation in poorly controlled patients living with diabetes, a new associated factor in the pathophysiology of Charcot’s acute neuroarthropathy
Source: PLoS One. 2020 May 21;15(5):e0233168. doi: 10.1371/journal.pone.0233168 (PMC7241699; doi:10.1371/journal.pone.0233168)
Supplement: S1 Table — (DOCX) [file pone.0233168.s004.docx]

**Supplementary Table 1-Therapeutic aspects of the included patients**

|  | T1DM (n=15) | T2DM (n=29) |
| --- | --- | --- |
| Intensification of antidiabetic treatment  *Insulin*  *Liraglutide*  *OADs^§^* | 10  -  - | 6  4  6 |
| Pancreas transplant | 1 |  |
| No treatment intensification | 1 | 9 |
| Unknown treatment intensification | 3 | 4 |

Values are given as number of patients. ^§^Oral antidiabetics.
