# Supplementary material for: Rapid glycemic regulation in poorly controlled patients living with diabetes, a new associated factor in the pathophysiology of Charcot’s acute neuroarthropathy
Source: PLoS One. 2020 May 21;15(5):e0233168. doi: 10.1371/journal.pone.0233168 (PMC7241699; doi:10.1371/journal.pone.0233168)
Supplement: S1 Image — (DOCX) [file pone.0233168.s005.docx]

**S1 Image: Flow chart of collecting data**

Radiology departements of

Centre hospitalier sud Francilien,

Hôpitaux Universitaires Pitié Salpêtrière - Charles Foix and

Centre hospitalier universitaire de Cochin

Screening RMI files with OAN en active phase

**59 files selected**

Check of inclusion criteria

15 excludes patients 44 included patients
